# Russell Body Esophagitis: A Possible Indication to Screen for Hematologic Malignancy

**DOI:** 10.7759/cureus.26291

**Published:** 2022-06-24

**Authors:** Glenn E Garcia, Muhammed R Hiba, Joseph Staffetti

**Affiliations:** 1 Internal Medicine, HCA Florida Bayonet Point Hospital, Hudson, USA; 2 Gastroenterology and Hepatology, HCA Florida Oak Hill Hospital, Brooksville, USA; 3 Gastroenterology, HCA Florida Bayonet Point Hospital, Hudson, USA

**Keywords:** oncology, esophagitis, russell body, pathology, gastroenterology, endoscopy

## Abstract

This is a case of an elderly man with lymphoplasmacytic lymphoma on a direct oral anticoagulant for atrial fibrillation who presented with weakness. Esophagogastroduodenoscopy found herpes esophagitis and islands of salmon-colored mucosa suspicious for Barrett’s esophagus. Biopsies showed no signs of Barrett’s Esophagus but returned positive for Russell bodies. This is the only reported case of Russell body esophagitis in the absence of Barrett’s esophagus. This case adds to the mounting evidence that Russell body esophagitis and potentially all gastrointestinal Russell bodies should prompt further work-up for hematologic malignancy.

## Introduction

Russell body esophagitis (RBE) was first described in the literature in 2005 [1]. The diagnosis is made via the identification of Russell bodies (RBs) within plasma cells on biopsies of the esophageal mucosa. RBs are inclusions of immunoglobulins found within the cytoplasm of plasma cells. Although there are few published cases of RBE, it is believed to share its pathogenesis with other gastrointestinal manifestations of RB inclusions. RBE has been primarily associated with Barrett’s esophagus [1-4]. RB gastritis (RBG) [5] and RB duodenitis (RBD) [6] have significantly more published cases and an apparent association with *Helicobacter pylori* [7].

## Case presentation

**Figure 1:**
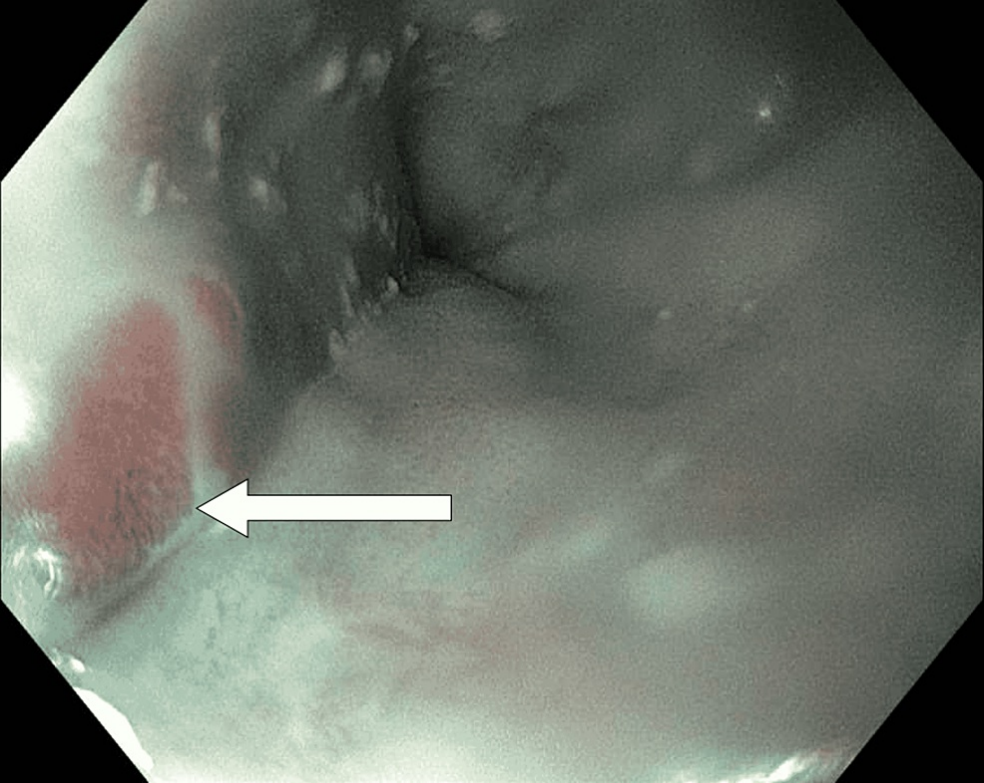
Salmon-colored mucosa visualized on esophagogastroduodenoscopy. Arrow indicates salmon-colored mucosa found to contain plasma cells with Russell bodies on histology. Biopsy confirmed that there was no evidence of Barrett's mucosa.

**Figure 2:**
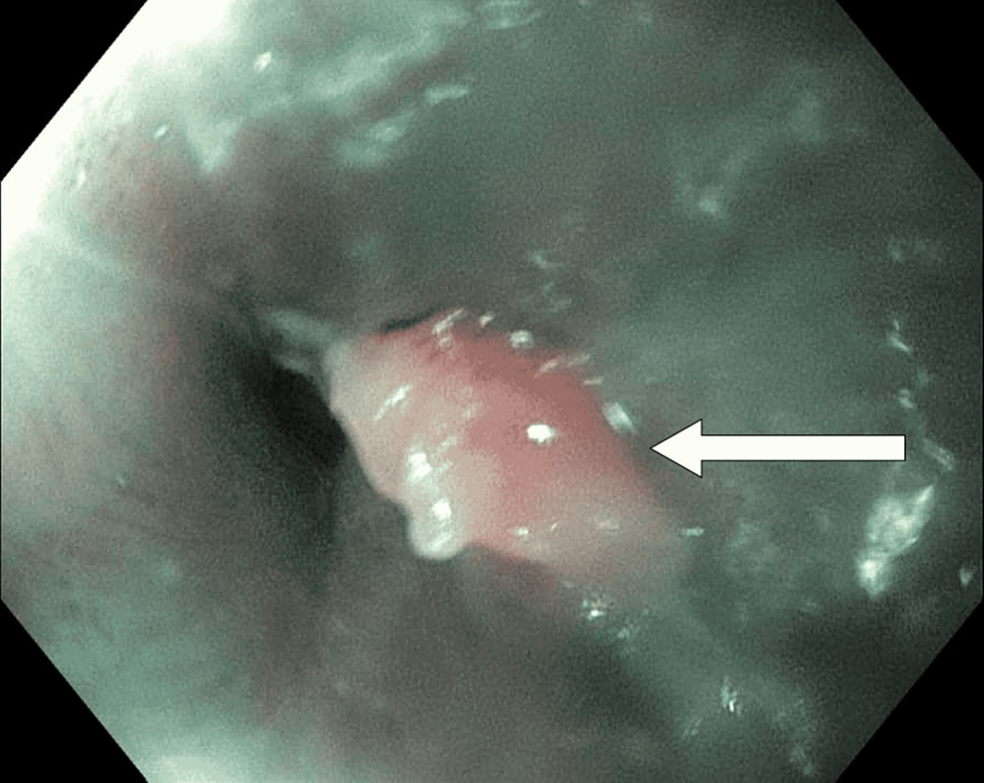
Nodule seen at gastroesophageal junction. Arrow indicates nodule confirmed via biopsy to be herpes esophagitis.

A Caucasian man over 80 years old with atrial fibrillation (on apixaban), chronic obstructive pulmonary disease, hypertension, hypothyroidism, lymphoplasmacytic lymphoma (LPL), and a history of upper gastrointestinal bleed was brought in by ambulance with a chief complaint of weakness. The patient was found to be anemic (hemoglobin 7.8 mg/dL), and Gastroenterology was consulted for further management. Esophagogastroduodenoscopy was performed. Islands of salmon-colored mucosa (Figure 1) were present at 35 cm. At the gastroesophageal junction (approximately 40 cm), a single 8 mm nodule was present (Figure 2). No source of upper gastrointestinal bleeding was identified. Biopsies of both lesions were taken, as well as gastric biopsies for *H. pylori*.

Biopsies were negative for Barrett’s esophagus. Biopsy of the esophageal nodule returned herpes esophagitis. Gastric biopsies revealed no evidence of *H. pylori*. 

**Figure 3:**
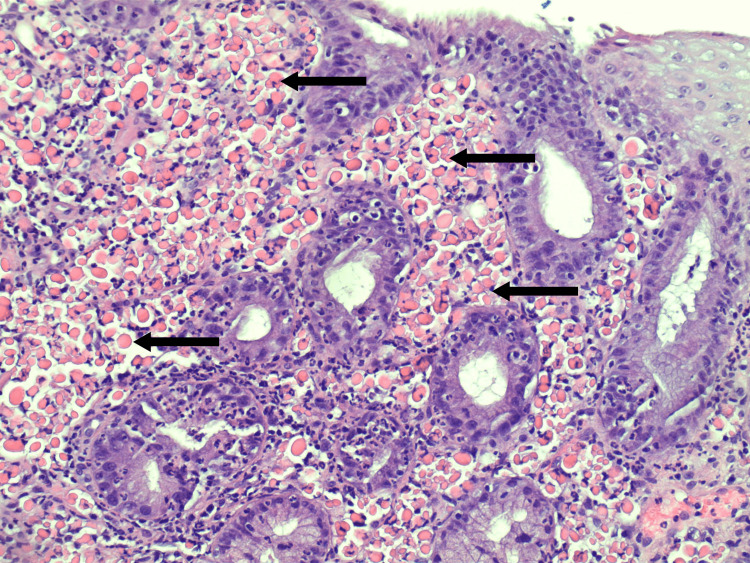
H&E stain demonstrating Russell bodies within B-cells in the lamina propria. Arrows indicate examples of positively staining cells. Medium power 20x magnification.

**Figure 4:**
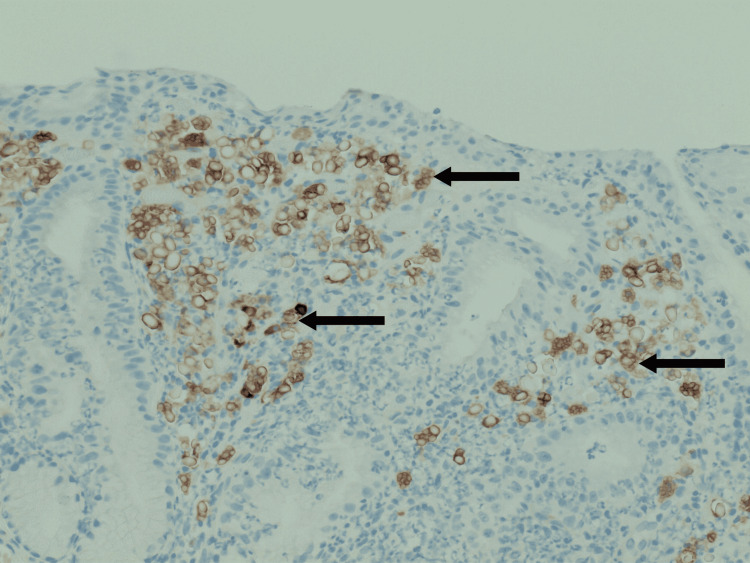
CD79a stain to confirm the presence of plasma cells with Russell bodies. Arrows indicate examples of positively staining cells. Medium power 20x magnification.

**Figure 5:**
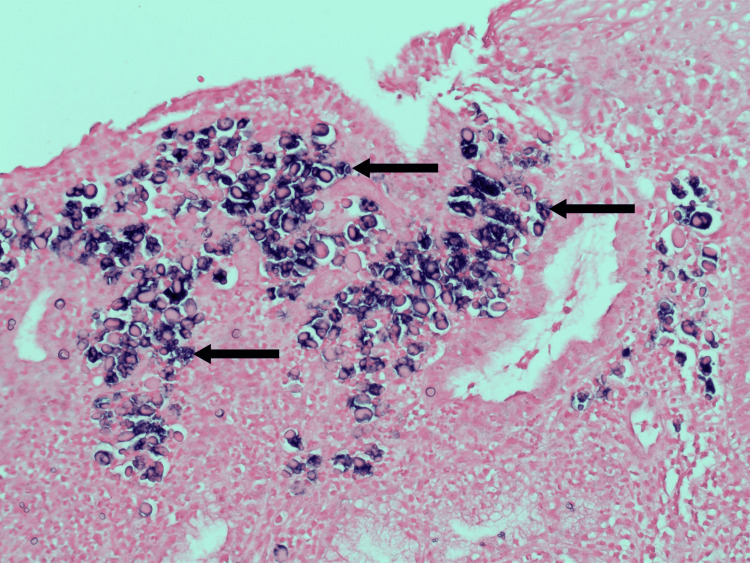
In situ hybridization for Immunoglobulin heavy chain and Kappa light chain. Arrows indicate examples of monoclonal plasma cells. Medium power 20x magnification.

H&E staining demonstrated pink globules within B-cells in the lamina propria (Figure 3). The CD138 and CD79a stains confirmed the presence of lymphocytes (Figure 4). In situ hybridization for the Kappa light chain showed clonal B lymphocytes (Figure 5). Additionally, clonal immunoglobulin heavy chain (IGH) and Kappa light chain (IGK) gene rearrangements were detected by PCR. Overall, the findings were compatible with RBE.

## Discussion

Given the association of gastrointestinal RBs with chronic inflammatory processes, the absence of the expected chronic gastrointestinal inflammatory processes should raise questions for further work-up. On endoscopy, this patient’s lesions appeared to be Barrett’s Esophagus; however, the pathology did not show any Barrett’s metaplasia, which makes this case of RBE unique and underscores the importance of endoscopic biopsy. RBG has been associated with malignancy, especially in the absence of *H. pylori *[8], but there are no reported cases of RBE and malignancy. Compared to most of the other reported cases of RBE in Table 1, our plasma cells with RBs were monoclonal for κ light chains and did not express λ light chains. LPL has been detected endoscopically in the stomach and the ileum [9-11]; LPL is classically known to cause the formation of monoclonal RBs in bone marrow [12]. Thus, the presence of RBs in the esophagus is likely a manifestation of this patient’s LPL. This case adds to the growing body of evidence that RBs in the upper gastrointestinal tract (in the absence of *H. pylori* and Barrett’s esophagus) herald malignancy.

**Table 1:** Published cases of Russell body esophagitis

Authors	Age/Sex	Biopsy Location	History	Endoscopy Findings	Histology/Immunology	H. pylori Infection	Barrett's Esophagus (Biopsy-confirmed)	Country
Rubio CA [1]	88/M	Esophagus	Gastroesophageal Reflux Disease	Barrett's esophagus	PAS stain, CD38, and CD138-positive plasma cells, with concomitant expression of κ and λ chains, containing Russell Bodies.	No	Positive	Sweden
Bhaijee F, Brown KA, Long BW, Brown AS [13]	69/M	Esophagus	Barrett's Esophagus, post-ablation	Residual band of Barrett's esophagus	PAS Stain and CD79a-positive plasma cells, with concomitant expression of κ and λ chains, containing Russell Bodies.	No	Positive	USA
Dhorajiya P, Mannan R [3]	82/M	Esophagus	Dysphagia	6 cm long Barrett’s mucosa	CD79a and CD138-positive plasma cells, with concomitant expression of κ and λ chains, containing Russell Bodies. Immunostaining for cytokeratin AE1/AE3 was negative.	No	Positive	USA
Arshi J, Nguyen J, Yin F [2]	41/M	Esophagus	Gastroesophageal Reflux Disease	Candida esophagitis, salmon colored mucosa in the distal esophagus	PAS stain and CD138-positive plasma cells containing Russell Bodies. Immunostaining for cytokeratin AE1/AE3 was negative	No	Positive	USA
Rangan A, Visscher DW [4]	80/M	Esophagus	Epigastric pain	Barrett's esophagus	CD138 positive plasma cells, with concomitant expression of κ and λ chains.	No	Positive	USA
Garcia, Hiba, Staffetti	91/M	Esophagus	Lymphoplasmacytic Lymphoma	Salmon-colored mucosa, Nodule at gastroesophageal junction	PAS stain, CD-138, and CD79a-positive plasma cells with Russell Bodies and expression of κ light chain.	No	Negative	USA

This case describes a new presentation of RBE - in the absence of Barrett’s esophagus. Our case of RBE comes in a patient with LPL and herpes esophagitis. Unlike LPL [10], herpes infection has not been reported to be associated with RBs in the gastrointestinal tract. The herpetic nodule may be noteworthy, finding given herpesviruses are a known source of RB cervicitis [14]. Owing to the dearth of cases, more research will be needed before herpesviruses can be considered causes of gastrointestinal RBs. In this case, the monoclonal plasma cells are more likely to have been caused by the patient's LPL. The authors posit that the herpes infection would more likely have led to polyclonal plasma cells (as in most of the other previously reported cases of RBE); however, more cases and research into the subject are needed.

## Conclusions

With only five published cases of RBE, the study of this histological finding continues to evolve. This case is unique because it is the only reported case of RBE in the absence of Barrett's esophagus. There is growing evidence for the association of gastrointestinal RBs with malignancies; however, a consensus has not yet been achieved. In this case, the patch of mucosa thought to be Barrett's esophagus turned out to be RBE. This is the first case of monoclonal RBE and the only case associated with malignancy (LPL). Although herpes esophagitis is a new association with RBE, the authors believe the monoclonal plasma cells are more indicative of LPL. Scientific understanding of gastrointestinal RB remains incomplete, and more cases will help to uncover the significance of this disease process.
